# Haemodynamic adaptations to isometric handgrip versus isometric wall squat exercise training: a randomised crossover study

**DOI:** 10.1007/s00421-026-06246-1

**Published:** 2026-04-22

**Authors:** Jamie J. Edwards, Andy Galbraith, Stewart Bruce-Low, Rajan Sharma, Jamie M. O’Driscoll

**Affiliations:** 1 Department of Applied Sport and Exercise Science, University of East London, London, UK; 2 Department of Cardiology, St George’s University Hospitals NHS Foundation Trust, London, UK; 3 Diabetes Research Centre, College of Life Sciences, University of Leicester, Leicester, UK; 4 Leicester Diabetes Centre, Leicester General Hospital, University Hospitals of Leicester NHS Trust, Leicester, UK

**Keywords:** Blood pressure, Hypertension, Isometric exercise training

## Abstract

**Purpose:**

Isometric exercise training (IET) has been shown to be an effective anti-hypertensive approach and there is a growing interest in the transition of IET into clinical exercise prescription settings. However, the most effective application of IET is not yet conclusive. As such, this work aimed to compare the haemodynamic effects of the two most prominent IET modes, the isometric wall squat (IWS) and isometric handgrip (IHG).

**Methods:**

In a cross-over design, 21 healthy participants were randomised to a 4-week IWS or IHG intervention, followed by a 4-week ‘washout’ period and then subsequent allocation to the opposite trial arm. Resting systolic (sBP) and diastolic (dBP) blood pressure, and other clinically important haemodynamic variables including heart rate (HR), stroke volume (SV), cardiac output (Q̇) and total peripheral resistance (TPR) were measured before and after the two 4-week study periods.

**Results:**

Following the intervention, resting clinic sBP, mBP and dBP were reduced in the IWS group (-6.8 mmHg, -5.5 mmHg and -4.8 mmHg) and IHG group (-4.8 mmHg, -3.2 mmHg and -2.5 mmHg). Comparatively, there was no significant condition x time interaction for sBP (β =  − 2.1 ± 1.3 mmHg, p = 0.102). Conversely, significantly greater reductions were observed for mBP (β =  − 2.3 ± 0.8 mmHg, p = 0.008) and dBP (β =  − 2.4 ± 1.1 mmHg, p = 0.025) following IWS compared with IHG.

**Conclusion:**

Overall, this study supports the utility of both isometric exercise training approaches in reducing blood pressure. While greater reductions in mBP and dBP were observed following IWS, both IWS and IHG represent effective and clinically relevant options for blood pressure management.

**Graphical Abstract:**

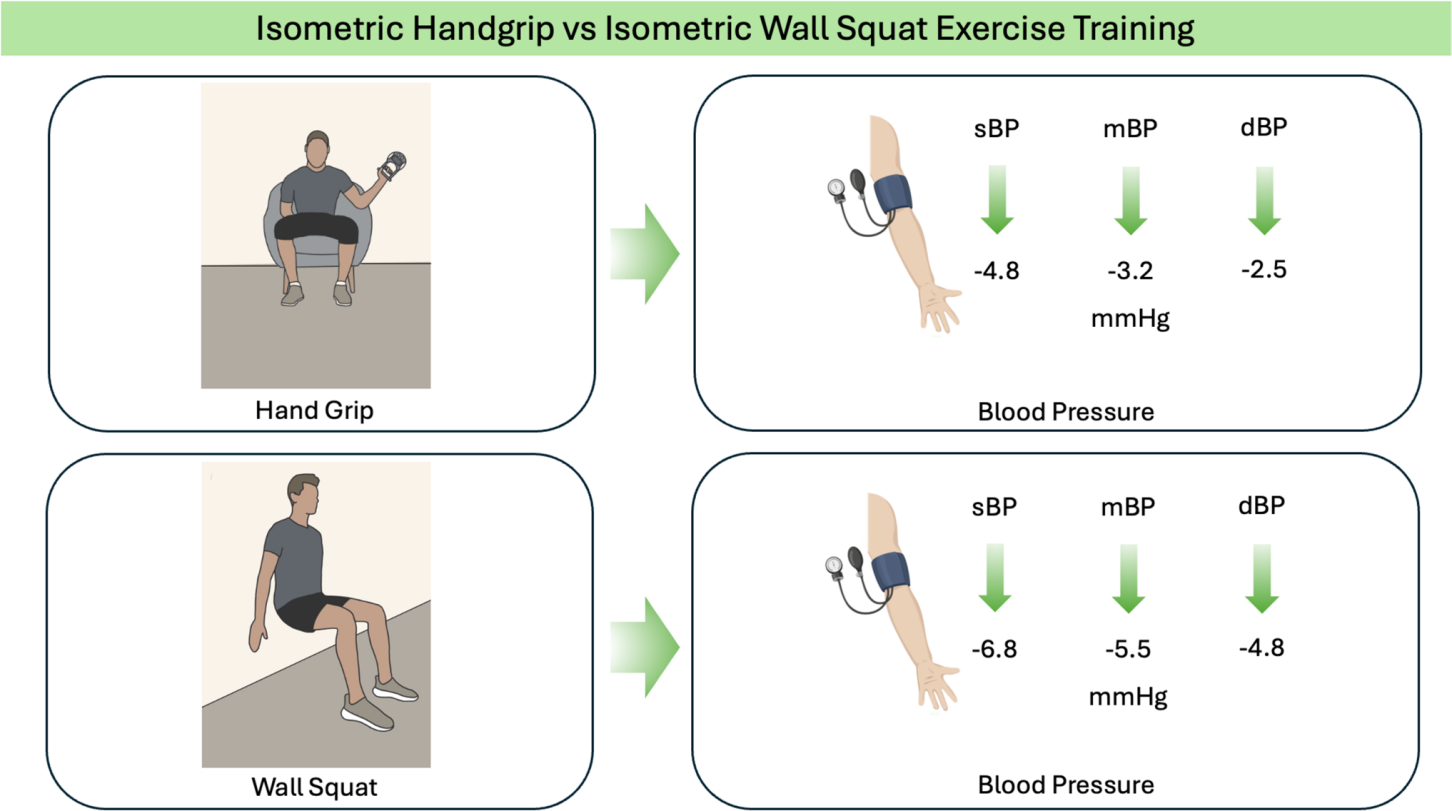

**Supplementary Information:**

The online version contains supplementary material available at 10.1007/s00421-026-06246-1.

## Introduction

Hypertension is closely linked to cardiovascular disease and mortality risk (Staplin et al. 2023), making effective management essential. A recent large-scale systematic review and network meta-analysis demonstrated that all modes of exercise produce statistically significant reductions in resting BP (Edwards et al. 2023). Of interest, isometric exercise training (IET), which is a convenient and time-efficient novel exercise approach, appears to produce the greatest magnitude of reduction with systolic and diastolic BP of 8 and 4 mmHg respectively, ranking 1st on the comparative surface under the cumulative ranking curve analysis (Edwards et al. 2023). Importantly, reductions of this magnitude are comparable to that seen with standard dose anti-hypertensive monotherapy (Law et al. 2009) and, if sustained, are likely to translate into improved cardiovascular outcomes (Ettehad et al. 2016).

Given this, there is a growing interest in the transition of IET into clinical exercise prescription settings; however, the most effective application of IET is not yet conclusive. Secondary analysis of this network meta-analysis subsequently compared the sub-modes of IET, finding the wall squat (IWS) to produce the greatest magnitude of change, although not to statistical significance, as is supported by an earlier systematic review and meta-analysis (Edwards et al. 2022b). There is also a physiological rationale for increased reductions with the IWS, with a greater extent of muscle mass recruited and thus surface area of vasculature compressed, aswell as the incorporation of postural and stabilising muscles (Edwards et al. 2022b, 2024). Despite the utility of previous comparative analyses, these are indirect and based on a small sample of heterogenous studies, highlighting the necessity for direct head-to-head randomised trial data to better understanding the comparative effects. Establishing the comparative effectiveness of these two sub-modes is important for clinical practice and exercise programming in understanding the interchangeability of their prescription, with the IWS being preferred by some, but more mobility-dependant.

As such, this study aimed to compare to the two most prominent IET modes, the IWS and the isometric handgrip (IHG). Specifically, the objectives of this study were to investigate the chronic haemodynamic adaptations following a 4-week intervention of IWS versus IHG exercise in a randomised cross-over design trial. We hypothesise that both the IWS and IHG will produce clinically significant reductions in resting systolic (sBP), mean (mBP) and diastolic (dBP) BP, with the IWS producing significantly greater reductions than that seen with the IHG.

## Methodology

### Participants and ethical approval

This study was a single centre, prospective randomised cross-over trial. Participants were considered eligible if they were healthy as defined by no significant self-reported health diagnoses, a normal 12-lead electrocardiogram and not currently taking any cardiovascular/anti-hypertensive medication. Participants were also required to be self-reported as physically inactive as defined by not meeting the current exercise guideline recommendations, non-smokers and free from injury or illness that may influence their ability to complete the intervention, affect the results, or jeopardise their well-being (Bull et al. 2020). This study included participants with normal to high normal BP according to the ESC/ESH guidelines (sBP < 140 mmHg and dBP < 90 mmHg) (Williams et al. 2018).

Written informed consent was obtained from all participants before testing and this study conformed to the Declaration of Helsinki principles with approval from the Canterbury Christ Church University Ethics Committee (ETH2021-0220).

### Study procedures

**Fig. 1:**
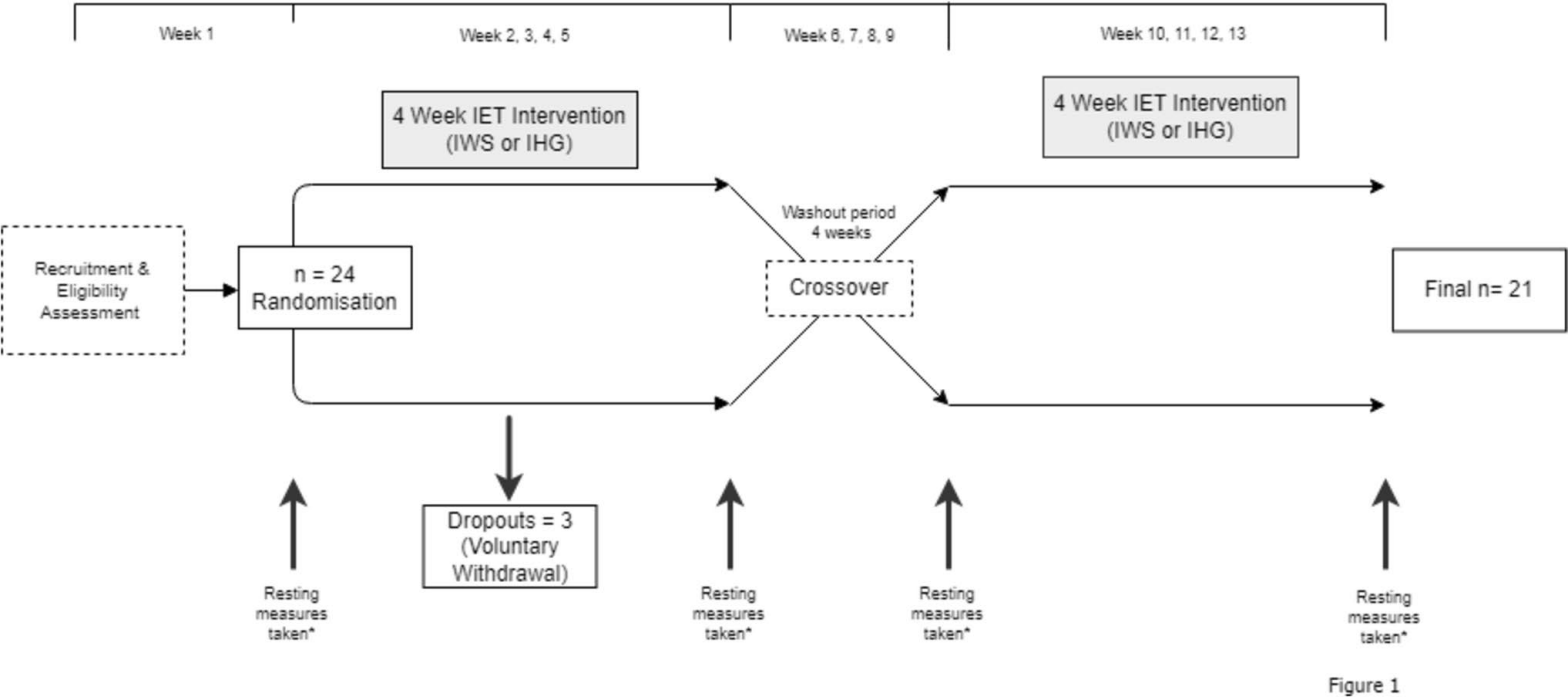
Study flow diagram depicting the randomised crossover design and measurement timepoints. *Indicates the measurement timepoint for resting systolic, mean and diastolic blood pressure and continuous haemodynamics

Eligible participants were randomly allocated to a 4-week IWS or IHG intervention, followed by a 4-week ‘washout’ period and then subsequent allocation to the opposite arm. Figure 1 presents a study flow diagram illustrating the study design with participant drop-outs. Participants were required to attend the lab on at least 5 occasions for before and after measures following both interventions. All participants were required to avoid alcohol and strenuous physical activity for 24 h, caffeine for 12 h and fast for at least 4 h prior to any lab testing. Furthermore, all participants were instructed to maintain normal circadian and dietary routines throughout the study period.

### Clinic blood pressure and haemodynamics

Resting clinic sBP and dBP were measured using a Dinamap Pro 200 device (Critikon; GE Medical Systems, Freiburg, Germany) in accordance with the current guidelines (Williams et al. 2018). As per the guidelines, both arms were measured, and the higher value arm was used as the reference (Williams et al. 2018). The participants had back support, an appropriate cuff size selected, and the arm was positioned at heart-level, with measurements taking in a seated position (Williams et al. 2018). Measures were taken before and after both the IWS and IHG interventions in a temperature-controlled room at the same time of the day. mBP was subsequently calculated automatically via the formula mBP = dBP + [1/3 x (sBP-dBP)].

All other haemodynamic measures were acquired via the Task Force Monitor (TFM), which is a validated non-invasive cardiovascular monitoring system that provides beat-to-beat data. Specifically, the TFM includes a six-channel electrocardiogram by which resting heart rate (HR) was measured. Stroke volume (SV) was acquired through impedance cardiography where three electrode bands were positioned on the participants, two of which were in line with the xiphoid process and adjacent to the thorax and the other placed on the nape of the neck. Cardiac output (Q̇) was subsequently auto calculated from HR and SV. Total peripheral resistance (TPR) was independently calculated according to Ohm’s law. Height and weight were acquired from all participants which was used to index Q̇ (Q̇I) and TPR (TPRI) to body surface area. All continuous measures were taken in a supine position after 15 min of rest. The haemodynamic parameters reported in this study are the average of a 5-min continuous recording.

### Isometric exercise training

**Fig. 2:**
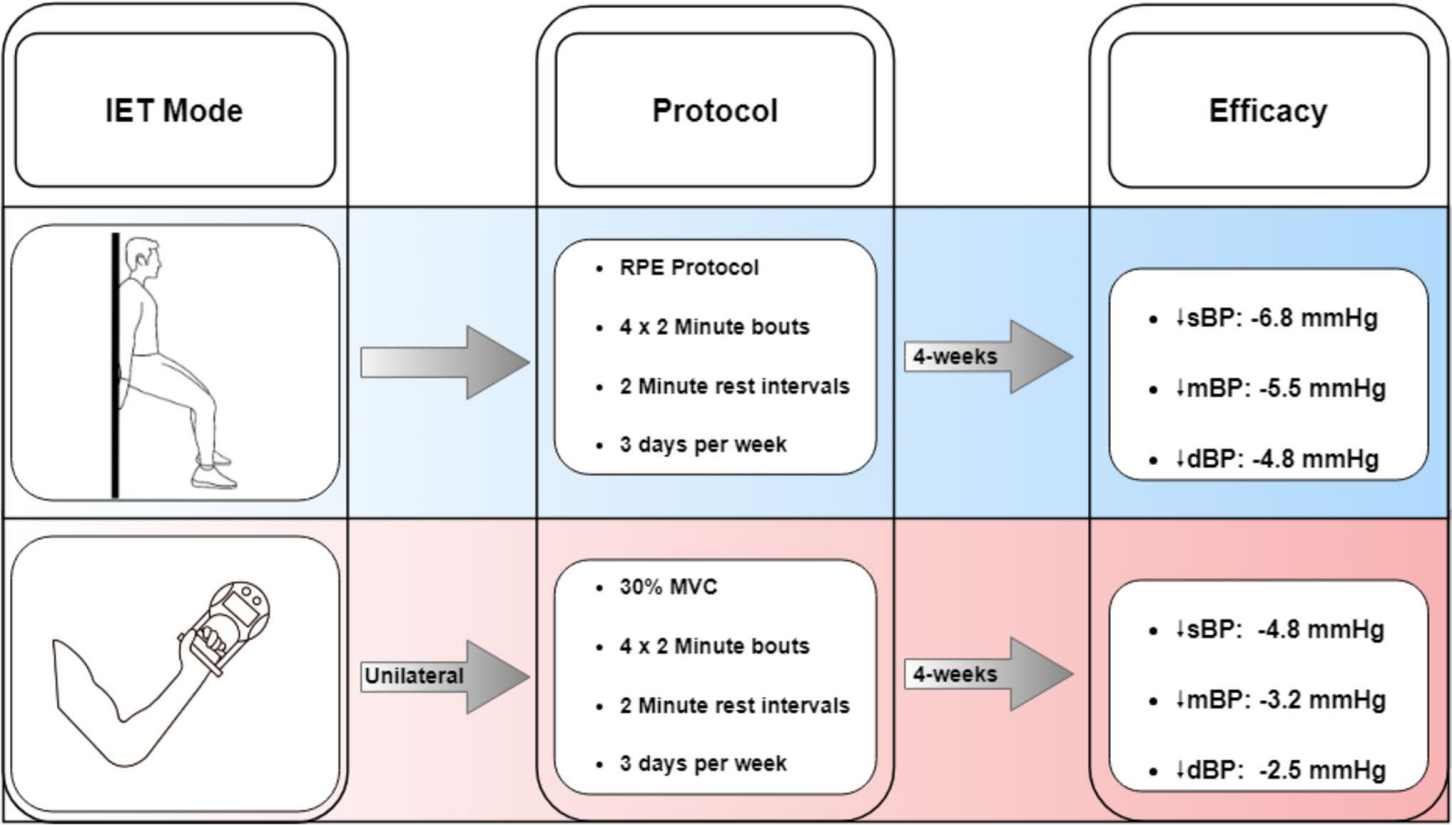
Modes, protocol details and efficacy of the isometric exercise training. *RPE* rate of perceived exertion; *MVC* maximum voluntary contraction; *sBP* systolic blood pressure; *mBP* mean blood pressure; *dBP* diastolic blood pressure

Figure 2 provides a graphical representation of the IET protocols employed for this study. All IET in this study was prescribed 3 × per week for 4 weeks (12 total sessions) in an unsupervised home-based setting via a training manual. Participants were encouraged to perform the IET with at least 48 h between sessions at the same time of the day. Each session followed a standard protocol of 4 × 2-min bouts, separated with 2-min rest intervals, resulting in a total session duration of 14-min. The IWS and IHG prescription intensity was individualised on their baseline visits. The IWS was prescribed according to the rate of perceived exertion (RPE) protocol as previously detailed (Lea et al. 2021a, 2023). In short, all participants performed a 30 s IWS, adjusting squat height and foot position (ensuring their lower legs always remained vertical) to find a position that they estimated were to elicit an RPE score of 4 on the Isometric Exercise Scale after a 2-min bout. The participant-selected squat height was then marked on the wall at the lowest point of contact, and this was used as the prescribed squat height.

The IHG prescription in this study followed a standardised protocol of 30% maximum voluntary contraction (MVC). To determine this MVC, the participants completed three maximal contractions using their non-dominant hand (the same hand they performed the weekly training with) taking the highest of the three efforts as their MVC. 30% of this value was then calculated as their prescribed training intensity. The non-dominant arm was selected to ensure consistency with the previous IHG literature (Edwards et al. 2022b, 2024). All IHG was performed using a programmed dynamometer with a digital display to show intensity (DHD-3 Digital Hand Dynamometer, Saehan Corp, South Korea). The participants were required to continuously monitor intensity throughout each bout, consciously trying to keep at the prescribed grip intensity. IHG prescription in this work followed a unilateral protocol to best reflect the most commonly applied protocols (Carlson et al. 2016; Gordon et al. 2018; Correia et al. 2020; Fecchio et al. 2023).

All participants were required to record their RPE score at the end of each IET bout during home-based training, which was monitored by the research team and compared to the target RPE zones. RPE was measured according to the Isometric Exercise Scale which is a modified derivative of the well-established CR-10 RPE scale as previously validated (Borg 1998). Target RPE values and corresponding zones were; bout 1: RPE 4 (3.5–4.5), bout 2: RPE 5.5 (5–6), bout 3: RPE 7 (6.5–7.5), and bout 4: RPE 8.5 (8–9) (Lea et al. 2021a, b, 2023). If their RPE score fell outside of the target zone for a bout, the participant was instructed to change the squat height by 1–2 cm at a time. As an important part of IET prescription, all participants were instructed to maintain normal breathing patterns during the IE contractions to avoid the haemodynamic changes associated with the Valsalva manoeuvre (Nóbrega et al. 1994). Adherence to the exercise was monitored through a home diary where participants were required to provide the details of each exercise session, including RPE intensity per-bout. The home diary was assessed by researchers following the completion of intervention.

### Sample size

The sample size for this study was based on the changes observed in previous 4-week intervention studies of similar IWS and IHG interventions in healthy populations (Carlson et al. 2016; Taylor et al. 2019). These studies have reported changes of 12 and 7 mmHg respectively for sBP, demonstrating a 5 mmHg change difference between IWS and IHG for sBP in these different, independent groups. This is considered clinically significant and represents a likely change difference of 4.2%. The sample size for this study was calculated using the Hopkins method (Hopkins 2000), which is based on measurement error and likely/worthwhile changes (alpha 0.05 and power of 80%). Using the CoV described in Wiles et al. (Wiles et al. 2010) (4.6%), the powered sample size for this cross-over design was 19 for sBP. Given the anticipated dropout rates with exercise training studies, we calculated a final sample size target of 24.

### Statistical analysis

All statistical analyses were performed using the Statistical Package for Social Sciences (SPSS version 29; SPSS Inc., Chicago, IL, USA). Continuous variables are presented as mean ± standard error of the mean unless otherwise stated.

To examine haemodynamic responses to the two exercise conditions, linear mixed-effects models were used. Condition (isometric handgrip vs wall-sit), time (pre- vs post-exercise), sequence (exercise order), and period (first vs second visit) were included as fixed effects, along with the condition x time interaction, which represented the primary treatment effect.

To account for repeated measurements within individuals, participant was included as a random intercept, thereby accounting for within-subject correlation. Models were estimated using restricted maximum likelihood with Satterthwaite approximation for degrees of freedom.

The crossover design was addressed by including sequence and period effects within the models to account for potential order and visit effects. The primary outcome of interest was the condition x time interaction, representing the difference in pre-to-post change between exercise conditions. Model results are reported as fixed-effect estimates with associated F statistics and p values. Statistical significance was accepted at p < 0.05.

## Results

Twenty-one participants (9 male, 12 female) completed the full study with 3 voluntary dropouts occurring throughout the study (Fig. 1). One participant dropped out due to family reasons, while the remaining 2 participants did not provide a reason. There were no significant between or within group differences from the pre-intervention measures and the post-washout measures, supporting the previously established notion that a 4-week washout period is sufficient for BP to return to baseline (Wiley et al. 1992; Howden et al. 2002). There were no adverse events during the IET interventions, and adherence was > 85% for all participants. As seen in Table 1, the mean age of the participants was 33 ± 14.1 years. With a mean height and weight of 171 ± 10.3 cm and 76 ± 12.7 kg, the mean BMI was 26 ± 4.4 kg/m^2^.

**Table 1:** Baseline Participant Demographics and Clinical Characteristics

Baseline characteristic	Isometric exercise training
*Sex (%)*	
Males	9 (43)
Females	12 (57)
Age (years)	33 ± 14.1
Height (cm)	171 ± 10.3
Weight (kg)	76 ± 12.7
BMI (kg/m^2^)	26 ± 4.4
*Ethnicity (%)*	
Asian	2 (10)
White	19 (90)

### Resting clinic blood pressure

Following the 4-week intervention, resting clinic BP decreased in both exercise conditions. In the IWS condition, systolic, mean, and diastolic BP decreased by − 6.8, − 5.5, and − 4.8 mmHg, respectively, while reductions of − 4.8, − 3.2, and − 2.5 mmHg were observed in the IHG condition.

**Fig. 3:**
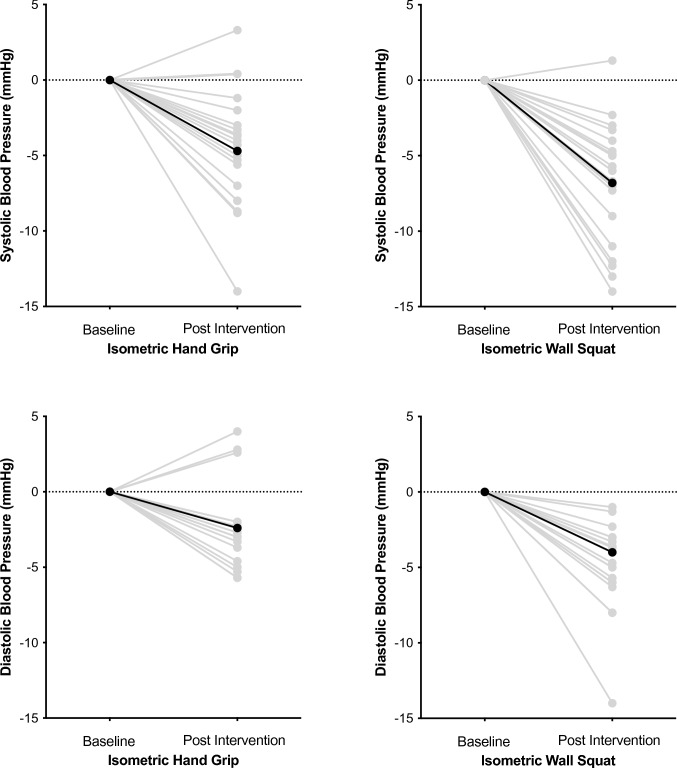
Individual participant changes in systolic (plots A and B) and diastolic (plots C and D) blood pressure following isometric handgrip and isometric wall squat exercise respectively

Linear mixed-effects models accounting for condition, time, sequence, and period, with participant included as a random intercept, demonstrated a significant condition x time interaction for mBP (F₁,₅₉ = 7.57, p = 0.008) and dBP (F₁,₅₉ = 5.27, p = 0.025), indicating greater reductions following the IWS compared with the IHG condition. However, the condition x time interaction for sBP was not significant (F₁,₅₉ = 2.75, p = 0.102), indicating that the magnitude of sBP reduction did not differ between exercise modalities. A significant sequence effect was observed for sBP (F₁,₁₉ = 11.07, p = 0.004), whereas no significant sequence effects were detected for mean or diastolic BP (all p > 0.05). No significant period effects were observed for systolic, mean, or diastolic blood pressure (all p > 0.05). Table 2 presents the primary condition x time interaction effects from the linear mixed models, while the full model outputs, including condition, time, sequence and period effects, are presented in Supplementary Table 1. Figure 3 illustrates the individual participant changes in systolic and diastolic blood pressure following isometric handgrip and isometric wall-sit exercise.

**Table 2:** Linear mixed model results for haemodynamic outcomes

Outcome	Effect	β	SE	F (df)	P value
Systolic blood pressure (mmHg)	Condition × Time	2.11	1.27	2.75 (1,59)	0.102
Mean blood pressure (mmHg)	Condition × Time	2.31	0.84	7.57 (1,59)	0.008
Diastolic blood pressure (mmHg)	Condition × Time	2.41	1.05	5.27 (1,59)	0.025
Heart rate (b⋅min^−1^)	Condition × Time	-0.79	2.03	0.15 (1,59)	0.698
Stroke volume (ml)	Condition × Time	-3.49	2.22	2.48 (1,59)	0.121
Stroke index (ml⋅m^2^)	Condition × Time	-1.77	1.1	2.58 (1,59)	0.114
Cardiac output (L⋅min^−1^)	Condition × Time	-0.34	0.24	1.97 (1,59)	0.165
Cardiac index (L⋅min^−1^⋅m^2^)	Condition × Time	-0.17	0.13	1.90 (1,59)	0.173
TPR (dyne⋅s⋅m^−5^)	Condition × Time	85.23	57.97	2.16 (1,59)	0.147
TPRI (dyne⋅s⋅m^−5^⋅m^2^)	Condition × Time	45.75	30.48	2.25 (1,59)	0.139

P-values represent the condition × time interaction from linear mixed-effects models including condition, time, sequence, and period as fixed effects and participant as a random intercept to account for within-subject correlation

*TPR* total peripheral resistance, *TPRI* total peripheral resistance index

### Haemodynamics

Haemodynamic responses were also analysed using linear mixed-effects models accounting for condition, time, sequence, and period effects. Heart rate decreased following both the IWS and IHG interventions; however, the condition x time interaction was not significant (F₁,₅₉ = 0.15, p = 0.698), indicating no difference between exercise conditions. Stroke volume increased following both interventions, but the condition x time interaction was not significant (F₁,₅₉ = 2.48, p = 0.121). Similarly, stroke index increased in both conditions with no significant difference between conditions (F₁,₅₉ = 2.58, p = 0.114). Cardiac output and cardiac index also increased following both interventions; however, no significant condition x time interactions were observed for cardiac output (F₁,₅₉ = 1.97, p = 0.165) or cardiac index (F₁,₅₉ = 1.90, p = 0.173). Total peripheral resistance and total peripheral resistance index decreased following both interventions, but no significant condition x time interactions were observed for TPR (F₁,₅₉ = 2.16, p = 0.147) or TPRI (F₁,₅₉ = 2.25, p = 0.139). Significant main effects of time were observed for several haemodynamic variables (Table 2), indicating physiological changes following both exercise interventions.

## Discussion

This study provides direct head-to-head randomised crossover design evidence of the comparative effectiveness of the IWS and IHG. We observed significant condition x time interactions for mBP and dBP, indicating greater reductions following the IWS intervention compared with IHG, while no interaction was observed for sBP. As illustrated in Fig. 2, this work demonstrates the effectiveness of both approaches in producing clinically meaningful changes and supports the utility of both approaches in reducing BP.

This study observed reductions in sBP, mBP and dBP of 6.8 mmHg, 5.5 mmHg and -4.8 mmHg following the IWS intervention, and 4.8 mmHg, 3.2 m mHg and 2.5 mmHg following the IHG intervention, respectively. While this work did not include a non-intervention control group to compare against, these changes are widely considered clinically significant and are generally in line with changes expected in healthy normotensive-pre-hypertensive individuals (Edwards et al. 2022b, a, 2023). In particular, a large-scale individual participant-level meta-analysis demonstrated a sBP reduction of 5 mmHg to lower the relative risk of major cardiovascular events by 10% (Adler et al. 2021). Until recently, there has been no robust evidence to support the superiority of one IET mode. However, considering the different stimuli, such as muscle mass, characteristics of activated muscles, and posture between IWS and IHG, it has been long hypothesised that clinically relevant response differences exist. Previous indirect data from pooled network meta-analyses showed reductions in sBP of 105/7.1 mmHg and dBP reductions of 5.3/3.5 mmHg for the IWS and IHG, respectively (Edwards et al. 2023). The lower magnitude of reduction observed in this work may be attributed to the normal/pre-hypertensive baseline BP of this cohort, whereas this pooled meta-analysis data also included studies of hypertensive cohorts.

To our knowledge only one other study has ever directly compared the effects of an IWS and IHG intervention in a randomised trial setting (Cohen et al. 2023). Cohen et al. (Cohen et al. 2023) compared BP changes following an IWS and IHG intervention in a group of unmedicated hypertensives, again evidencing larger mean reductions of 12.9/11.9 mmHg sBP and 4.1/4 mmHg dBP. Interestingly, this study found no significant difference in the magnitude of sBP change between the two modes. Contrasting differences between the two studies include the research design (crossover vs independent groups), difference in intervention duration (4 vs 12 weeks), wall squat prescription and baseline BP inclusion criteria. Cohen et al. (Cohen et al. 2023) also provided sessional exercise supervision, differing from the home-based exercise prescription in the present study. While it is unclear the degree to which each of these differences moderated the magnitude of BP change following the interventions, the general take-home message of this study and the wider literature remains similar with both modes representing effective options.

The mechanistic changes driving reductions in BP following IET are complicated and still largely unclear. With little or no change in Q̇, it appears that adaptations to the vascular system, which ultimately produce a reduction in TPR are key. Of which, IET has been previously associated with improvements in locally regulated conduit and resistance vessel endothelial-dependent vasodilation, functional adaptations in autonomic vasomotor control, and systemically modulated structural vascular remodelling (Edwards et al. 2022b, 2024). In healthy adults, these TPR changes have been closely linked with improvements in autonomic cardiovascular regulation with corresponding changes in HRV metrics, although the discussion of population-specific mechanistic underpinnings are still largely hypothetical in nature (Edwards et al. 2022b, 2024). Comparatively, the greater magnitude of effect on mBP and dBP seen with the IWS is probably attributable to differences in the extent of recruited muscle mass and thus surface area of compressed vasculature when compared to IHG protocols (Millar et al. 2014), while the incorporation of postural and stabilising muscles when holding the squat position may be an important distinguishing factor from leg extension IET (Wiles et al. 2010). Indeed, previous research from Swift et al. (Swift et al. 2022) compared the acute responses, evidencing a substantially greater acute haemodynamic intra-exercise increase in (sBP, mBP, dBP, HR and Q̇), with a subsequent significantly larger post-exercise hypotensive response in recovery, mediated by a greater drop in TPR. As such, the results of this 4-week training study suggest that the transient increase in BP from acute exercise may translate into sustained reductions in cardiovascular hemodynamics from chronic IET training.

While the IWS appears to produce a greater magnitude of mBP and dBP reduction, it is also important to consider the wider clinical context as to the utility of the IHG separate from magnitude of reduction. The IHG is an important alternative in clinical populations who may struggle to complete the mobility demanding IWS, which carries an additional risk of falls and musculoskeletal injury. The application of the IWS may be limited or even contraindicated in a subgroup of patients who cannot safely hold an IWS position due to various reasons, such as knee pathology, obesity and lack of sufficient musculoskeletal fitness or general frailty-related mobility problems. Additionally, there is greater concern around the cardiovascular risk associated with the substantially greater acute haemodynamic responses to the IWS compared to the IHG, suggesting the IHG may be most suitable in more acutely vulnerable populations (Coneglian et al. 2023). The lack of statistical significance between both modes is likely an absence of superiority rather than true equivalence, although from a clinical relevance perspective emphasises the utility of both approaches. Overall, the practical application of these findings provides increased flexibility on IET prescription, driven by the clinical context and suitability.

### Limitations

Several limitations should be acknowledged when interpreting these findings. First, this study did not include a non-intervention control group; however, the primary aim was to directly compare two isometric exercise training modes using a randomised crossover design, thereby maximising statistical efficiency and reducing inter-individual variability. Second, the study was powered specifically to detect differences in blood pressure outcomes, limiting inference regarding mechanistic haemodynamic variables.

Participants were young, predominantly healthy adults with normal to high-normal blood pressure, which may limit generalisability to older or clinical hypertensive populations. Health status was primarily self-reported, which may introduce bias. Finally, the cohort was predominantly White British, highlighting the need for further research in more ethnically diverse populations.

## Conclusion

This head-to-head randomised crossover trial demonstrated no significant difference in sBP reductions between IET modes, although greater reductions in mBP and dBP were observed following IWS compared with IHG. Both interventions produced clinically meaningful reductions in blood pressure, supporting the utility of either IET modality for BP management. Future research should examine the effectiveness of these approaches in clinical populations.

## Supplementary Information

Below is the link to the electronic supplementary material.

Supplementary file1 (DOCX 19 KB)

## Data Availability

The datasets generated and/or analysed during the current study are available from the corresponding author on reasonable request.

## Abbreviations

BP: Blood pressure
IET: Isometric exercise training
IHG: Isometric handgrip
IWS: Isometric wall squat
Q̇: Cardiac output
dBP: Diastolic blood pressure
HR: Heart rate
MVC: Maximum voluntary contraction
mBP: Mean blood pressure
RPE: Rate of perceived exertion
sBP: Systolic blood pressure
SV: Stroke volume
TFM: Task force monitor
TPR: Total peripheral resistance
