# Supplementary material for: Haemodynamic adaptations to isometric handgrip versus isometric wall squat exercise training: a randomised crossover study
Source: Eur J Appl Physiol. 2026 Apr 22;126(7):4205–14. doi: 10.1007/s00421-026-06246-1 (PMC13380598; doi:10.1007/s00421-026-06246-1)
Supplement: Supplementary file 1 — Supplementary file1 (DOCX 19 KB) [file 421_2026_6246_MOESM1_ESM.docx]

| Supplemental Table 1: Linear mixed model results for haemodynamic outcomes | | | |
| --- | --- | --- | --- |
| Parameter | Effect | F (df) | P |
| sBP (mmHg) | Condition | 0.37 (1,59) | 0.544 |
|  | Time | 83.35 (1,59) | <0.001 |
|  | Sequence | 11.07 (1,19) | 0.004 |
|  | Period | 1.80 (1,59) | 0.184 |
|  | Condition × Time | 2.75 (1,59) | 0.102 |
| mBP (mmHg) | Condition | 0.32 (1,59) | 0.576 |
|  | Time | 108.08 (1,59) | <0.001 |
|  | Sequence | 3.83 (1,19) | 0.065 |
|  | Period | 2.84 (1,59) | 0.097 |
|  | Condition × Time | 7.57 (1,59) | 0.008 |
| dBP (mmHg) | Condition | 0.09 (1,59) | 0.761 |
|  | Time | 48.31 (1,59) | <0.001 |
|  | Sequence | 1.47 (1,19) | 0.24 |
|  | Period | 1.46 (1,59) | 0.231 |
|  | Condition × Time | 5.27 (1,59) | 0.025 |
| HR (b⋅min^-1^) | Condition | 0.91 (1,59) | 0.344 |
|  | Time | 3.61 (1,59) | 0.062 |
|  | Sequence | 0.90 (1,19) | 0.354 |
|  | Period | 0.55 (1,59) | 0.46 |
|  | Condition × Time | 0.15 (1,59) | 0.698 |
| SV (ml) | Condition | 2.29 (1,59) | 0.136 |
|  | Time | 53.31 (1,59) | <0.001 |
|  | Sequence | 1.64 (1,19) | 0.216 |
|  | Period | 0.03 (1,59) | 0.866 |
|  | Condition × Time | 2.48 (1,59) | 0.121 |
| SI (ml⋅m^2^) | Condition | 3.10 (1,59) | 0.083 |
|  | Time | 61.28 (1,59) | <0.001 |
|  | Sequence | 0.21 (1,19) | 0.65 |
|  | Period | 0.01 (1,59) | 0.905 |
|  | Condition × Time | 2.58 (1,59) | 0.114 |
| Q̇ (L⋅min^-1^) | Condition | 2.52 (1,59) | 0.118 |
|  | Time | 11.11 (1,59) | 0.001 |
|  | Sequence | 0.32 (1,19) | 0.578 |
|  | Period | 0.50 (1,59) | 0.484 |
|  | Condition × Time | 1.97 (1,59) | 0.165 |
| Q̇I (L⋅min^-1^⋅m^2^) | Condition | 2.50 (1,59) | 0.119 |
|  | Time | 11.23 (1,59) | 0.001 |
|  | Sequence | 0.01 (1,19) | 0.923 |
|  | Period | 0.28 (1,59) | 0.6 |
|  | Condition × Time | 1.90 (1,59) | 0.173 |
| TPR (dyne⋅s⋅m^-5^) | Condition | 4.17 (1,59) | 0.046 |
|  | Time | 27.85 (1,59) | <0.001 |
|  | Sequence | 0.16 (1,19) | 0.696 |
|  | Period | 0.09 (1,59) | 0.763 |
|  | Condition × Time | 2.16 (1,59) | 0.147 |
| TPRI (dyne⋅s⋅m^-5^⋅m^2^) | Condition | 3.68 (1,59) | 0.06 |
|  | Time | 29.22 (1,59) | <0.001 |
|  | Sequence | 0.001 (1,19) | 0.971 |
|  | Period | 0.07 (1,59) | 0.789 |
|  | Condition × Time | 2.25 (1,59) | 0.139 |

Note: sBP, systolic blood pressure; mBP, mean blood pressure; dBP, diastolic blood pressure; HR, heart rate; SV, stroke volume; SI, stroke index; Q̇, cardiac output; Q̇I, cardiac index; TPR, total peripheral resistance; TPRI, TPR index. P-values represent fixed effects from linear mixed-effects models including condition, time, sequence, and period as fixed factors with participant included as a random intercept.
